# Knowledge, attitudes, and practices of community pharmacists regarding the management of diabetes during Ramadan in the Aseer Region, Saudi Arabia

**DOI:** 10.3389/fphar.2026.1827330

**Published:** 2026-06-15

**Authors:** Moteb Khobrani

**Affiliations:** Clinical Pharmacy Department, College of Pharmacy, King Khalid University, Abha, Saudi Arabia

**Keywords:** attitudes, community pharmacists, diabetes mellitus, knowledge, practices, ramadan, Saudi Arabia

## Abstract

**Background:**

Fasting during Ramadan causes challenges for diabetes treatment, necessitating tailored counseling and adjustments for medication. Community pharmacists play a vital role in enhancing fasting safety, although their readiness has not been well investigated in southern Saudi Arabia. This study aimed to evaluate community pharmacists’ knowledge, attitudes, and practices (KAP) regarding diabetes management during Ramadan in the Aseer Region, and to identify perceived barriers and the need for additional training.

**Methods:**

A descriptive cross-sectional study was performed from February to May 2025 with 301 licensed community pharmacists in the Aseer Region. Participants were recruited through professional pharmacy social media platforms and pharmacy communication groups commonly used within the region. Data were collected utilizing a previously validated self-administered questionnaire. Descriptive and inferential statistics were conducted using SPSS v26, with a significance level set at p < 0.05. Inferential findings were interpreted cautiously as subgroup comparisons.

**Results:**

A total of 301 pharmacists participated. Knowledge of key safety measures during fasting was high, with 91.7% correctly identifying the blood glucose threshold (<60 mg/dL) requiring termination of fasting. Correct recognition of high-risk patients who should avoid fasting was reported for patients with recurrent hypoglycemia (88.7%) and elderly or unwell patients (87.4%). Knowledge related to medication regimen adjustment was lower, particularly in insulin dose modification, where 71.1% provided correct responses. In practice, more than 80% counseled patients on blood glucose monitoring and meal planning, while 63.8% addressed physical activity adjustments, and 68.1% counseled on medication regimen changes. The most frequently reported barriers were time constraints (85.7%), lack of updated training (84.7%), and inadequate counseling privacy (79.8%). Prior training was associated with higher knowledge and practice scores (p = 0.001). Pharmacists who attended training workshops demonstrated higher mean knowledge and practice scores than untrained pharmacists.

**Conclusion:**

Community pharmacists in the Aseer Region demonstrated generally adequate knowledge and positive attitudes regarding diabetes management during Ramadan, particularly in fasting-safety counseling and patient education. However, important gaps remained in advanced medication regimen adjustment practices, especially insulin dose modification, as well as participation in structured public education activities. Continuing professional development programs focused on Ramadan-specific diabetes management and interprofessional collaboration may improve pharmacist-led diabetes care during Ramadan.

## Introduction

Fasting in the month of Ramadan constitutes one of the five pillars of Islam and is practiced by over two billion Muslims worldwide (World Population Review, 2023). This month, Muslims refrain from consuming food, beverages, and oral medications from dawn till sunset for a duration of 29–30 days (Deniz et al., 2025). Despite Islam’s exemption for individuals with chronic illnesses, a significant number of diabetes patients opt to fast; the Epidemiology of Diabetes and Ramadan (EPIDIAR) study indicated that 78.7% of type 2 diabetes patients fasted, resulting in a 7.5-fold increased risk of severe hypoglycemia compared to non-fasting days (Salt et al., 2004). The fasting during Ramadan presents numerous challenges in sustaining glycemic control, adhering to medication regimens, and preserving hydration, underscoring the necessity for culturally customized pharmaceutical care (Deniz et al., 2025).

Community pharmacists are pivotal in integrating clinical and cultural elements of diabetes management during Ramadan. Pharmacists’ accessibility designates them as the primary point of contact for pharmaceutical counseling, glucose monitoring guidance, and personalized fasting risk assessment (Erku et al., 2017). A literature review by Almansour et al. indicated that pharmacists are both eager and able to assist fasting patients with diabetes; however, they necessitate further training in clinical and cultural competency (Almansour et al., 2017). In Saudi Arabia, where diabetes prevalence exceeds 18% (Sun et al., 2022), and Ramadan fasting is practically ubiquitous among Muslim adults, community pharmacists can substantially impact safe fasting practices through medication regimen adjustments (MRA) and patient education.

Despite this potential, research conducted in many countries has uncovered deficiencies in pharmacists’ readiness. Amin and Chewning found that only 16% of Egyptian pharmacists proactively engaged in Ramadan-related medication discussions, citing this to insufficient confidence and absence of official guidance (Amin and Chewning, 2014). Abdelaziz et al. in Sudan indicated that 75% of pharmacists accurately recognized high-risk patients, although more than 70% attributed a lack of expertise as a barrier to counseling (Abdelaziz et al., 2019). Studies from Jordan and other international pharmacy-practice settings have similarly reported variability in pharmacists’ preparedness, counseling practices, and confidence regarding diabetes management during Ramadan (Almomani et al., 2025; Adnan et al., 2016; Wilbur et al., 2014). These data suggest that although pharmacists recognize the need for diabetes management tailored to Ramadan, organized teaching initiatives are limited.

Recent evidence underscores the worldwide necessity for culturally competent healthcare provision. Hillier et al. emphasized that healthcare workers in Western nations frequently possess insufficient understanding of fasting physiology, religious exemptions, and cultural norms, resulting in discomfort and inadequate communication with Muslim patients (Hillier et al., 2023). Addressing these deficiencies through focused professional development improves patient safety and trust. In Saudi Arabia, these findings correspond with national healthcare transformation goals outlined in Vision 2030, which aim to empower pharmacists as essential members of multidisciplinary primary care teams.

From a clinical standpoint, managing diabetes during Ramadan requires careful adjustment of insulin and oral antidiabetic medications. Almalki and Alshahrani reviewed options for managing type 2 diabetes during fasting, highlighting the importance of personalized pre-Ramadan evaluations, dosage adjustments, and continuous glucose monitoring to avert hypoglycemia and dehydration (Almalki and Alshahrani, 2016). The IDF-DAR Practical Guidelines endorse pharmacist-led education, individualized pre-fasting risk assessment, and multidisciplinary support for patients with diabetes during Ramadan (International Diabetes Federation Diabetes and Ramadan IDF-DAR International Alliance, 2021; Hassanein et al., 2022). Considering these global and national priorities, evaluating community pharmacists’ knowledge and perspectives on Ramadan-specific diabetes management is essential. Although previous studies have explored pharmacists’ roles in Ramadan diabetes management in countries such as Egypt, Sudan, Jordan, and Pakistan, limited region-specific evidence is available regarding community pharmacists’ preparedness, counseling practices, perceived barriers, and Ramadan-focused diabetes training within the Aseer region of Saudi Arabia. Therefore, this study was conducted to provide region-specific evidence from the Aseer region rather than to establish conceptual novelty of the research topic itself. This study aims to evaluate community pharmacists’ knowledge, attitudes, and behaviors about diabetes treatment during Ramadan, identify perceived obstacles to effective counseling, and investigate the potential for capacity enhancement. The results of this study will guide ongoing educational programs and enhance evidence-based approaches that support the Vision 2030 objective of optimizing pharmacy-based primary care in Saudi Arabia.

## Methods

### Study design and setting

A descriptive, cross-sectional study was performed among community pharmacists in the Aseer region of Saudi Arabia from February to May 2025. The study focused on pharmacists employed in independent and chain community pharmacies. This design was selected to evaluate pharmacists’ knowledge, attitudes, and practices (KAP) for diabetes care during Ramadan in a practical setting.

### Study population and sampling

The study involved 301 licensed community pharmacists, selected via convenience sampling from major urban areas and surrounding regions. Participants were recruited through professional pharmacy social media platforms and pharmacy communication groups commonly used by community pharmacists in the Aseer region. Eligibility was verified using self-reported screening questions confirming that respondents were licensed community pharmacists, currently practicing in community pharmacies, and residing in the Aseer region at the time of survey completion. Responses were obtained from pharmacists practicing across different cities and surrounding areas within the Aseer region.

To minimize duplicate entries, the online survey platform (Google Forms) was configured to allow only platform-based restrictions (e.g., single-submission settings), and participants were instructed to complete the questionnaire only once. Incomplete questionnaires and responses not meeting eligibility criteria were excluded before analysis. Because the survey link was distributed openly through social media channels, the total number of pharmacists who received the invitation could not be determined; therefore, a response rate could not be calculated, and the denominator was considered unknown.

The present sample of 301 pharmacists was considered a pragmatic sample size for an exploratory cross-sectional study. No formal *a priori* power calculation was performed. Therefore, inferential analyses were interpreted as exploratory group-level comparisons rather than confirmatory analyses of determinants or predictors of KAP outcomes. The sample size was considered sufficient for descriptive estimation of proportions and preliminary subgroup comparisons within the study population. The sample size also falls within the range commonly considered adequate for descriptive cross-sectional surveys among healthcare professionals.

The inclusion criteria included licensed pharmacists with a minimum of 6 months of community practice experience who voluntarily consented to participate. Interns, hospital pharmacists, and incomplete responses were excluded.

### Instrument development

The survey instrument was modified from the validated questionnaire created by Abdelaziz et al., which evaluated pharmacists’ knowledge, attitudes, and practices for diabetes care during Ramadan in Sudan (Abdelaziz et al., 2019). The instrument was adapted to suit the Saudi setting by integrating elements consistent with the IDF-DAR Practical Guidelines (International Diabetes Federation Diabetes and Ramadan IDF-DAR International Alliance, 2021). Two reviewers with expertise in diabetes care and community pharmacy practice evaluated the adapted instrument. The adaptation and reporting process followed the step-by-step statistical and methodological framework outlined by Sayed (Sayed, 2024).

Questionnaire items were reviewed for relevance, clarity, cultural appropriateness, and suitability for community pharmacy practice in Saudi Arabia. Minor wording modifications were made based on reviewer feedback. A pilot assessment for questionnaire clarity and readability was conducted before final dissemination of the survey. However, no formal content validity index was calculated.

The final instrument consisted of five sections (World Population Review, 2023): demographic data (Deniz et al., 2025), knowledge (Salt et al., 2004), practice (Erku et al., 2017), attitude, and (Almansour et al., 2017) perceived barriers.

### Scoring and categorization

The knowledge domain consisted of 10 items assessing risk stratification, criteria for terminating fasting, and medication regimen adjustment. Each item was scored as 1 for a correct response and 0 for incorrect or “unsure” responses, yielding a total possible knowledge score ranging from 0 to 10. Because the knowledge items assessed different factual aspects of diabetes management during Ramadan, knowledge findings were interpreted primarily at the item level rather than as a single internally correlated construct. Accordingly, internal consistency measures such as Cronbach’s alpha were considered less appropriate for the knowledge domain because the items were not intended to measure a single latent construct.

The practice domain included 7 items measured using a three-point Likert scale (Always = 3, Sometimes = 2, Never = 1), with total practice scores ranging from 7 to 21. Practice levels were categorized as high (≥75% of the total score), moderate (50%–74%), and low (<50%).

The attitude and perceived barriers domains were assessed using five-point Likert scales (Strongly agree to Strongly disagree). Higher scores reflected more positive attitudes and stronger perceived barriers, respectively.

### Data collection

Data were gathered via online self-administered questionnaires disseminated via professional pharmacy social media platforms. Participation was both voluntary and anonymous. The completion of the survey implied informed consent.

### Data analysis

Data were processed utilizing IBM SPSS Statistics version 26. Descriptive statistics, including frequencies, percentages, means, standard deviations, and selected 95% confidence intervals, summarize participant attributes and responses. The statistical analysis followed a step-wise analytical approach adapted from Sayed (2024), beginning with descriptive analyses of demographic variables and questionnaire responses, followed by inferential comparisons across demographic subgroups (Sayed, 2024).

Inferential approaches, including Chi-square tests, independent-samples t tests, and one-way ANOVA, were utilized to examine the relationships between demographic characteristics and KAP scores. Independent-samples t tests were applied for two-group comparisons, whereas one-way ANOVA was used for variables containing more than two categories. Assumptions of normality and homogeneity of variance were assessed before inferential testing. Confidence intervals were calculated for selected two-group comparisons where sufficient summary data were available. Post hoc subgroup analyses and additional multivariable analyses were not performed. Therefore, significant ANOVA findings should be interpreted as preliminary group-level associations. A p-value less than 0.05 was considered statistically significant.

The questionnaire used in this study was adapted from a previously validated instrument, for which content validity and internal consistency had been established in earlier research. In the present study, additional internal consistency testing was not re-evaluated in the present study. Therefore, the interpretation of total domain scores was performed cautiously. Analyses were limited to descriptive and inferential statistical procedures based on collected responses.

### Ethical considerations

Ethical approval was obtained from the Research Ethics Committee of the College of Pharmacy, King Khalid University (approval no. KKU-110–2025–30). No personal information was collected, and the results were utilized exclusively for research reasons. All data were collected anonymously, stored in password-protected electronic files, and accessed only by the research team to ensure confidentiality and data security throughout the study.

## Results

### Socio-demographic characteristics of participants

The survey included 301 community pharmacists. Male (61.1%) and non-Saudi (59.1%) responders were predominant, with Saudi pharmacists making up 40.9%. Nearly half of the participants (48.2%) were aged 26–30, followed by 21–25 (21.9%), 31–35 (18.9%), and 36–40 (10%). Only 3 participants (1.0%) were older than 40 years.

Most pharmacists (73.1%) had Pharm. D degrees, while 23.9% had B. Pharm and 3% had postgraduate degrees. Regarding experience, 56.2% had practiced for less than 5 years, 28.9% five to 10 years, and 14.9% more than 10 years. Most pharmacists (81.4%) worked in chain pharmacies, 18.6% in independents. One-third (32.6%) had attended diabetes or Ramadan management workshops. Table 1 outlines participant sociodemographics.

**TABLE 1:** Sociodemographic characteristics of community pharmacists (n = 301).

Variable	Category	Frequency (n)	Percentage (%)
Gender	Male	184	61.1
	Female	117	38.9
Nationality	Saudi	123	40.9
	Non-saudi	178	59.1
Age group (years)	21–25	66	21.9
	26–30	145	48.2
	31–35	57	18.9
	36–40	30	10.0
	>40	3	1.0
Educational qualification	B.Pharm	72	23.9
	Pharm.D	220	73.1
	Higher education (Master’s/Ph.D.)	9	3.0
Years of experience	<5 years	169	56.2
	5–10 years	87	28.9
	>10 years	45	14.9
Type of pharmacy	Independent	56	18.6
	Chain	245	81.4
Attended training/workshop on diabetes or Ramadan management	Yes	98	32.6
	No	203	67.4

### Knowledge of diabetes management during ramadan

Table 2 summarizes the knowledge of participants towards diabetes management during Ramadan. Most participants demonstrated generally adequate responses across several knowledge items related to diabetes management during Ramadan, with mean accurate responses above 80% across various domains. High-risk patients with recurrent hypoglycemia (88.7%) and elderly or unwell patients (87.4%) should avoid fasting. When blood glucose drops below 60 mg/dL, 91.7% know to stop fasting, and 85.7% know to stop when it reaches 300 mg/dL.

**TABLE 2:** Knowledge of diabetes management during ramadan among community pharmacists.

Knowledge item	Correct n (%)	Incorrect n (%)	Unsure n (%)
Risk categorization			
1. A well-controlled diabetic patient on metformin is at low risk	246 (81.7)	28 (9.3)	27 (9.0)
2. A well-controlled diabetic on sulfonylurea is at moderate risk	218 (72.4)	47 (15.6)	36 (12.0)
3. Patients with recurrent hypoglycemia should not fast	267 (88.7)	19 (6.3)	15 (5.0)
4. Elderly or ill diabetic patients should avoid fasting	263 (87.4)	22 (7.3)	16 (5.3)
When to terminate fasting			
5. Fasting should stop if blood glucose <60 mg/dL	276 (91.7)	11 (3.7)	14 (4.6)
6. Fasting should stop if blood glucose is ≤70 mg/dL in the morning	252 (83.7)	23 (7.6)	26 (8.6)
7. Fasting should stop if blood glucose >300 mg/dL	258 (85.7)	17 (5.6)	26 (8.6)
Medication regimen adjustment (MRA)			
8. Adjusting metformin 500 mg TID to 1,000 mg Iftar +500 mg suhoor	235 (78.1)	33 (10.9)	33 (10.9)
9. A larger oral dose should align with the main meal (Iftar)	245 (81.4)	29 (9.6)	27 (9.0)
10. Insulin dose should be shifted to Iftar and reduced at suhoor	214 (71.1)	51 (16.9)	36 (12.0)

Medication regimen adjustment (MRA) knowledge was lower but acceptable, with 78.1% appropriately altering the metformin dose and 81.4% timing oral medicine with Iftar. Knowledge related to insulin dose modification showed comparatively lower accuracy (71.1%). As the knowledge items addressed different factual components of diabetes management during Ramadan, the findings were interpreted primarily at the item level.

### Practice patterns toward diabetes management during ramadan among community pharmacists

Ramadan counseling was regular for most pharmacists (Table 3). Over 80% consistently advised diabetics to monitor blood glucose during fasting and plan meals to avoid hypoglycemia and dehydration. Similarly, 76.7% counseled patients on post-Iftar meal selection to prevent hyperglycemia.

**TABLE 3:** Practice patterns of community pharmacists toward diabetes management during ramadan.

Practice item	Always n (%)	Sometimes n (%)	Never n (%)
1. Advise diabetic patients to monitor blood glucose during fasting hours	247 (82.1)	45 (15.0)	9 (3.0)
2. Counsel patients on meal planning to prevent hypoglycemia and dehydration	238 (79.1)	52 (17.3)	11 (3.6)
3. Educate patients on selecting meals to avoid post-Iftar hyperglycemia	231 (76.7)	57 (18.9)	13 (4.3)
4. Recommend appropriate timing and intensity of physical activity	192 (63.8)	89 (29.6)	20 (6.6)
5. Provide counseling on medication regimen adjustment (MRA)	205 (68.1)	73 (24.3)	23 (7.6)
6. Refer high-risk patients to physicians before Ramadan	216 (71.8)	67 (22.3)	18 (6.0)
7. Participate in or organize public awareness or education campaigns	127 (42.2)	113 (37.5)	61 (20.3)

Fewer pharmacists routinely addressed physical activity adjustments (63.8%) or medication regimen changes (68.1%), and just 42.2% participated in diabetes and fasting public education. However, over 70% of participants refer high-risk patients, such as those with recurrent hypoglycemia, to doctors before Ramadan.

### Attitudes and perceived barriers toward community pharmacists’ professional role in diabetes management during ramadan

During Ramadan, pharmacists were mostly satisfied with their diabetes treatment roles. Over 94% agreed or strongly agreed that pharmacy practice requires medication regimen adjustment knowledge, and 93% agreed that pharmacists improved diabetic outcomes. Moderate confidence: 82.7% feel confident counseling fasting diabetes patients, while 89% wanted Ramadan-specific training. Most responders (90.6%) believed that physician collaboration improves diabetes care, indicating multidisciplinary involvement.

Most respondents highlighted time restrictions (85.7%), lack of updated training (84.7%), and inadequate counseling privacy (79.8%). Low patient engagement (79.4%) was another issue. Table 4 shows Ramadan attitudes and barriers to professional diabetes management.

**TABLE 4:** Attitudes and perceived barriers of community pharmacists toward diabetes management during ramadan.

Statement	Strongly agree n (%)	Agree n (%)	Neutral n (%)	Disagree n (%)	Strongly disagree n (%)
Attitudes					
1. Knowledge of medication regimen adjustment (MRA) is essential for pharmacy practice	194 (64.5)	89 (29.6)	14 (4.7)	3 (1.0)	1 (0.3)
2. Pharmacists play a key role in improving diabetes outcomes during Ramadan	186 (61.8)	94 (31.2)	17 (5.6)	3 (1.0)	1 (0.3)
3. I feel confident counseling diabetic patients about fasting and medication adjustments	128 (42.5)	121 (40.2)	39 (13.0)	10 (3.3)	3 (1.0)
4. I would like additional training on Ramadan-specific diabetes management	152 (50.5)	116 (38.5)	23 (7.6)	8 (2.7)	2 (0.7)
5. Collaboration with physicians improves the quality of diabetes care	166 (55.1)	107 (35.5)	22 (7.3)	5 (1.7)	1 (0.3)
Perceived barriers					
1. Lack of up-to-date knowledge or continuing training	142 (47.2)	113 (37.5)	30 (10.0)	11 (3.7)	5 (1.7)
2. Lack of privacy or a suitable counseling area	129 (42.9)	111 (36.9)	33 (11.0)	20 (6.6)	8 (2.7)
3. Time constraints and pharmacy workload	161 (53.5)	97 (32.2)	26 (8.6)	11 (3.7)	6 (2.0)
4. Low patient interest or adherence	128 (42.5)	111 (36.9)	41 (13.6)	15 (5.0)	6 (2.0)

### Correlation and inferential findings

Selected demographic variables showed statistically significant associations with specific KAP domains (Table 5). Subgroup comparisons suggested differences in mean knowledge and practice scores across age groups (p = 0.034). Differences in knowledge and attitude scores were also observed across educational levels (p = 0.022), and practice scores differed significantly according to the number of years of professional experience (p = 0.017).

**TABLE 5:** Relationship between demographic variables and KAP scores of community pharmacists.

Demographic variable	Knowledge (mean ± SD)	Attitude (mean ± SD)	Practice (mean ± SD)	Outcome(s) associated with reported test	Test/Statistic	p-value	95% CI
Gender	​	​	​	None	t = 1.27	0.205	−0.49 to 0.17
Male (n = 184)	8.12 ± 1.45	22.51 ± 2.71	19.87 ± 2.98	​	​	​	​
Female (n = 117)	8.28 ± 1.36	22.38 ± 2.77	19.28 ± 3.26	​	​	​	​
Age group (years)	​	​	​	Knowledge and Practice	F = 2.94	0.034*	—
21–25 (n = 66)	7.91 ± 1.52	21.96 ± 2.68	19.15 ± 3.22	​	​	​	​
26–30 (n = 145)	8.25 ± 1.39	22.67 ± 2.74	19.72 ± 3.08	​	​	​	​
31–35 (n = 57)	8.32 ± 1.41	22.89 ± 2.68	20.04 ± 2.92	​	​	​	​
36–40 (n = 30)	8.48 ± 1.37	22.63 ± 2.69	20.11 ± 2.97	​	​	​	​
>40 (n = 3)	8.60 ± 1.28	22.10 ± 2.61	19.87 ± 3.02	​	​	​	​
Educational qualification	​	​	​	Knowledge and Attitude	F = 3.88	0.022*	—
B.Pharm (n = 72)	7.92 ± 1.44	21.98 ± 2.83	19.12 ± 3.17	​	​	​	​
Pharm.D (n = 220)	8.29 ± 1.37	22.61 ± 2.69	19.74 ± 3.07	​	​	​	​
Higher education (n = 9)	8.45 ± 1.22	23.02 ± 2.58	20.31 ± 2.88	​	​	​	​
Years of experience	​	​	​	Practice	F = 4.12	0.017*	—
<5 years (n = 169)	8.02 ± 1.45	22.14 ± 2.79	19.48 ± 3.21	​	​	​	​
5–10 years (n = 87)	8.24 ± 1.38	22.56 ± 2.71	19.72 ± 3.12	​	​	​	​
>10 years (n = 45)	8.56 ± 1.27	22.93 ± 2.58	20.18 ± 2.94	​	​	​	​
Training attended	​	​	​	Knowledge and Practice	t = 3.22	0.001*	0.18 to 0.84
Yes (n = 98)	8.49 ± 1.32	22.93 ± 2.54	20.24 ± 2.91	​	​	​	​
No (n = 203)	7.98 ± 1.46	22.18 ± 2.82	19.33 ± 3.19	​	​	​	​

* Statistically significant at p < 0.05.

95% confidence intervals were calculated for the mean knowledge score differences in the gender and training attendance comparisons only.

Post hoc subgroup comparisons were not performed; therefore, ANOVA, findings should be interpreted cautiously.

Attending training was associated with trained pharmacists revealing greater knowledge (8.49 ± 1.32, p = 0.001) and practice scores (20.24 ± 2.91, p = 0.001) than untrained colleagues. The 95% confidence interval for the difference in knowledge scores between trained and untrained pharmacists ranged from 0.18 to 0.84. No statistically significant differences were observed across gender for any KAP domain.

## Discussion

This study provides region-specific evidence regarding community pharmacists’ knowledge, attitudes, and practices toward diabetes management during Ramadan in the Aseer Region of Saudi Arabia. The findings demonstrated generally adequate knowledge of key fasting safety measures, positive attitudes toward pharmacist-led counseling, and frequent involvement in patient education practices related to glucose monitoring and meal planning during Ramadan. However, important gaps remained in medication regimen adjustment, particularly insulin dose modification, as well as in participation in structured public education activities.

The present findings are generally consistent with previous studies conducted in Egypt, Sudan, Jordan, and other pharmacy-practice settings. A Jordanian study reported that 9.5% of pharmacists demonstrated excellent knowledge regarding diabetes management during Ramadan, whereas 72.3% had intermediate knowledge and 18.2% had inadequate knowledge, with approximately 58.1% reporting good practice levels (Almomani et al., 2025). Similarly, Sudanese pharmacists previously demonstrated adequate recognition of high-risk fasting patients and fasting termination criteria, whereas deficiencies in proactive Ramadan-related counseling have been reported among Egyptian pharmacists (Amin and Chewning, 2014; Abdelaziz et al., 2019). Comparable findings regarding pharmacist involvement in Ramadan-focused diabetes counseling and medication management have also been reported in other international pharmacy-practice settings (Adnan et al., 2016; Wilbur et al., 2014). In the current study, pharmacists demonstrated stronger performance in glucose-monitoring counseling and fasting-safety recommendations than in advanced medication-adjustment practices, particularly insulin modification. These observations are generally consistent with previous international findings rather than representing unique patterns specific to the Aseer Region, and they further support the need for structured, evidence-based Ramadan-focused diabetes education and counseling guidance for pharmacists (Hassanein et al., 2017).

A previous study reported that community pharmacists have a substantial impact on diabetes management, particularly through patient education. Wilbur et al. demonstrated that pharmacists play an important role in medication review, dose adjustment advice, and patient counseling during Ramadan (Wilbur et al., 2014). This current study revealed that pharmacists provided counseling services, including medication regimen adjustment and recommendations for several nutritional and vitamin supplements to individuals with diabetes. This aligns with findings from Deniz et al., who reported that patient-centered counseling delivered in community pharmacies contributed positively to diabetes self-management during Ramadan (Deniz et al., 2025).

Patient education remains an essential component of diabetes care during Ramadan because it can improve medication adherence, self-monitoring practices, glycemic control, and patients’ understanding of fasting-related risks. Recent evidence from Saudi Arabia demonstrated that structured diabetes education was associated with improved diabetes control and reduced complications among adult patients with diabetes (Al-Nozha et al., 2024). In the present study, most pharmacists routinely provided counseling regarding blood glucose monitoring, meal planning, and fasting safety, suggesting that community pharmacists may contribute meaningfully to patient education when appropriate training and practice support are available. However, this study did not directly evaluate patient outcomes or medication adherence; therefore, any clinical impact should be interpreted cautiously.

The findings of the current study indicate that pharmaceutical counseling services for individuals with diabetes may help improve patients’ self-management skills by offering information on medication use, diabetes management, and lifestyle modifications. This observation is supported by evidence showing that pharmacist-led diabetes care is both clinically beneficial and cost-effective (Zhu et al., 2024). In addition, Standards of Care in Diabetes emphasize individualized treatment adjustment and patient education as key components of diabetes management (American Diabetes Association Professional Practice Committee, 2024), areas where community pharmacists can contribute meaningfully when adequately trained.

Additionally, recent research from Saudi Arabia suggests that pharmacy organizations should implement systematic, standardized, and comprehensive education programs for diabetes to enhance pharmacists’ proficiency in managing chronic diseases (Rasheed et al., 2023; Thorakkattil et al., 2024; Al Rifai et al., 2022). The current study conducted in the Aseer Region highlights that community pharmacists recognize the need for ongoing, practice-oriented training to enhance their ability to counsel and monitor fasting patients with diabetes. Participation in these programs would improve clinical skills and support the establishment of pharmacist-led diabetic services in the community, as demonstrated in prospective evaluations of integrated clinical pharmacy services (Kutluay et al., 2025). Although the majority of pharmacists in this study demonstrated adequate general knowledge, the findings revealed shortcomings in specialized areas, such as insulin regimen modification and personalized counseling, a finding also reported among community pharmacists in the Aseer region (Salem et al., 2025). These findings may reflect the need for more structured continuing professional development opportunities focused on Ramadan-specific diabetes management.

The perceived barriers identified in this study, including time constraints, workload, limited counseling privacy, and lack of updated training, were also comparable to barriers reported in previous regional and international studies. These barriers may influence the consistency and depth of pharmacist-led counseling services during Ramadan. The findings therefore support ongoing efforts to strengthen practice environments, multidisciplinary collaboration, and continuing education opportunities for community pharmacists involved in diabetes care.

This study has several limitations. The cross-sectional design assesses pharmacists’ knowledge, attitudes, and practices at a specific moment, therefore failing to demonstrate causation or evaluate changes following educational interventions. Secondly, the data relied on self-administered questionnaires, potentially introducing reporting bias or social desirability bias, as pharmacists may have exaggerated their counseling procedures. Additionally, the use of convenience sampling through professional social media platforms may have introduced self-selection bias, as pharmacists with a greater interest in diabetes care or Ramadan counseling may have been more likely to participate. Because the denominator of eligible pharmacists was unknown, a response rate could not be calculated. Therefore, the representativeness and generalizability of the findings should be interpreted cautiously. Third, the study was limited to the Aseer Region; therefore, the results may not comprehensively reflect community pharmacists in other regions of Saudi Arabia with varying demographic or professional attributes. The study lacked direct observation of counseling behavior or patient outcomes, which could have offered objective assessments of practice quality and clinical efficacy. In addition, although the questionnaire was adapted from a previously validated instrument, internal consistency was not re-evaluated in the present study, and the knowledge items assessed different factual components of Ramadan-related diabetes management rather than a single internally correlated construct. Therefore, reliability measures such as Cronbach’s alpha may not have been fully appropriate for the knowledge domain. Furthermore, inferential comparisons were exploratory because no formal *a priori* power calculation was conducted, and additional multivariable analyses were beyond the scope of the current exploratory analysis. Ultimately, future research should include qualitative interviews and longitudinal follow-up to provide deeper insights into pharmacists’ experiences and to assess the efficacy of specialized training programs.

## Conclusion

This research presents region-specific evidence from the Aseer Region regarding the involvement of community pharmacists in the management of diabetes care throughout Ramadan. The results showed that pharmacists have a good understanding of the basics and a good attitude toward managing diabetes while fasting, especially when it comes to teaching patients and keeping an eye on their blood sugar levels. However, gaps remain in advanced skills such as identifying high-risk patients, adjusting insulin, and modifying medication regimens. Pharmacists face challenges, including limited time, workload, insufficient formal training, and barriers affecting the consistency of counseling services.

To address these issues, continuing professional development programs should be standardized and aligned with international guidelines for diabetes management during Ramadan. Enhancing pharmacist authority and promoting interprofessional collaboration may help strengthen patient safety and diabetes care. Future research should evaluate the clinical impact of pharmacist-led interventions using larger and more representative study populations. These findings may help inform future educational and pharmacy practice initiatives aimed at supporting safer fasting practices among patients with diabetes in the Aseer Region and other parts of Saudi Arabia.

## Data Availability

The original contributions presented in the study are included in the article/supplementary material, further inquiries can be directed to the corresponding author.
